# Supplementary material for: Synthesis, Characterization, and the Antioxidant Activity of Carboxymethyl Chitosan Derivatives Containing Thiourea Salts
Source: Polymers (Basel). 2019 Nov 4;11(11):1810. doi: 10.3390/polym11111810 (PMC6918197; doi:10.3390/polym11111810)
Supplement: Supplementary file 1 [file polymers-11-01810-s001.pdf]

Supporting Information

Significantly enhanced antioxidant activity of chitosan through chemical modification with thiourea salts

Table S1. Antioxidant activity comparison

Types of antioxidant activity	Sample	Antioxidant effect
DPPH radicals’ scavenging ability	The oligo-maltose fraction from Polygonum Cillinerve [2]	26.67% (5mg\ml)
	A new polysaccharide from Bletilla striata fibrous roots [1]	64.67% (5mg\ml)
	Different phenolic acids grafted onto chitosan[3]	80% (2mg\ml)
	Carboxymethyl chitosan derivatives containing thiourea salts	More than 80%, even up to 100% (1.6mg\ml)
Hydroxyl radicals’ scavenging activity	The oligo-maltose fraction from Polygonum Cillinerve [2]	91.83% (4.5mg\ml)
	Different phenolic acids grafted onto chitosan [3]	70% (2mg\ml)
	Carboxymethyl chitosan derivatives containing thiourea salts	More than 55%, even up to 100% (1.6mg\ml)
Superoxide radicals’ scavenging ability	A new polysaccharide from Bletilla striata fibrous roots [1]	72.27% (5mg\ml)
	Different phenolic acids grafted onto chitosan [3]	80% (2mg\ml)
	Carboxymethyl chitosan derivatives containing thiourea salts	More than 90%, even up to 100% (1.6mg\ml)

References	[1]. Z. Chen, Y. Zhao, X.Wei, Structural characterization and antioxidant activity of a new polysaccharide from Bletilla striata fibrous roots, *Carbohydrate Polymers,* 2019.115362<br><br>[2]. Y. Zhou, W. Ma, L. Wang, Y. Fan, Characterization and antioxidant activity of the oligo-maltose fraction from Polygonum Cillinerve, *Carbohydrate Polymers*, 2019, 12,15<br><br>[3]. Y. Wang, M. Xie, G. Ma, F. Pei, The antioxidant and antimicrobial activities of different phenolic acids grafted onto chitosan, *Carbohydrate Polymers*, 2019.12.01
